# Crystal structure of (2,2′-bi­pyridine-κ^2^ *N*,*N*′)-*trans*-bis­(*tert*-butyl­dimethyl­sil­yloxy)-*cis*-dioxidomolybdenum(VI)

**DOI:** 10.1107/S2056989018008472

**Published:** 2018-06-15

**Authors:** Mikhail E. Minyaev, Alexander A. Vinogradov, Ilya E. Nifant’ev, Andrei V. Churakov

**Affiliations:** a A.V. Topchiev Institute of Petrochemical Synthesis, Russian Academy of Sciences, 29 Leninsky Prospect, 119991, Moscow, Russian Federation; b Chemistry Department, M.V. Lomonosov Moscow State University, 1 Leninskie Gory Str., Building 3, Moscow 119991, Russian Federation; c N.S. Kurnakov Institute of General and Inorganic Chemistry, Russian Academy of Sciences, 31 Leninsky Prospect, Moscow 119991, Russian Federation

**Keywords:** crystal structure, molybdenum(VI), rare structural motif, coordination compounds, NMR, hydrogen bonding.

## Abstract

In the title compound, [(^*t*^BuSiMe_2_O)_2_MoO_2_(2,2′-bi­pyridine)] or [Mo(C_6_H_15_OSi)_2_O_2_(C_10_H_8_N_2_)], the Mo^VI^ atom has a distorted octa­hedral environment with the sil­oxy substituents occupying the *trans* positions. The complex contains a rare (*R*_3_SiO)_2_ *M*O_2_ (*M* = Mo, W) structural motif and was formed in a reaction between sodium molybdate and *tert*-butyl­dimethyl­silyl chloride in the presence of 2,2-bi­pyridine. In the crystal, neighbouring mol­ecules are linked by C—H⋯O=Mo hydrogen bonds, forming chains propagating along the *a*-axis direction.

## Abstract 2

In the title compound, [(^*t*^BuSiMe_2_O)_2_MoO_2_(2,2′-bi­pyridine)], the Mo^VI^ atom has a distorted octa­hedral environment with the sil­oxy substituents occupying the *trans* positions.

## Chemical context   

Bulky sil­oxy ligands are of inter­est as they can stabilize transition metal complexes with low coordination numbers, providing attractive structures and chemistry (Eppley *et al.*, 1991 ▸; Neithamer *et al.*, 1989 ▸; Huang & DeKock, 1993 ▸). The structural and reactivity studies of *cis*-*M* ^VI^O_2_ and *cis*-*M* ^VI^OS complexes (*M* = Mo, W), including sil­oxy derivatives, are essential for understanding the activity of specific enzymes (Thapper *et al.*, 1999 ▸; Miao *et al.*, 2000 ▸). Both Mo^VI^O_2_ and Mo sil­oxy derivatives have attracted attention as precursors, or as real catalytic species, in various catalytic applications (Heppekausen *et al.*, 2012 ▸; Arzoumanian *et al.*, 2008 ▸; Coelho *et al.*, 2011 ▸; Bruno *et al.*, 2006 ▸). Herein, we report on the crystal structure and synthesis of the title complex, (^t^BuSiMe_2_O)_2_MoO_2_(bipy) (I).
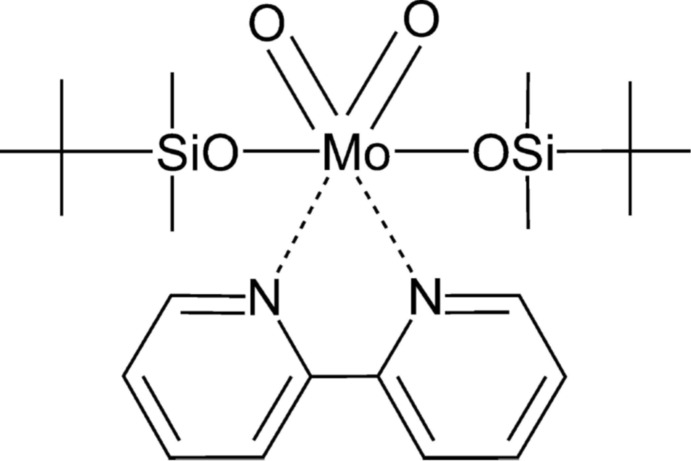


**Figure 1:**
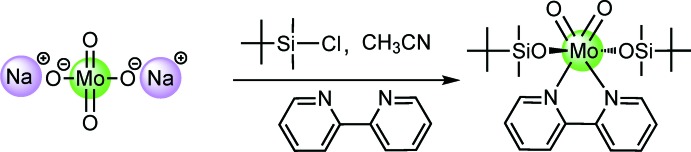
Synthesis of [(^*t*^BuSiMe_2_O)_2_MoO_2_(2,2′-bi­pyridine)] (I).

It was prepared by a one-pot reaction of sodium molybdate (Na_2_MoO_4_) with 2,2-bi­pyridine (bipy) in aceto­nitrile followed by addition of *tert*-butyl­dimethyl­silyl chloride (Fig. 1 ▸).

## Structural commentary   

**Figure 2:**
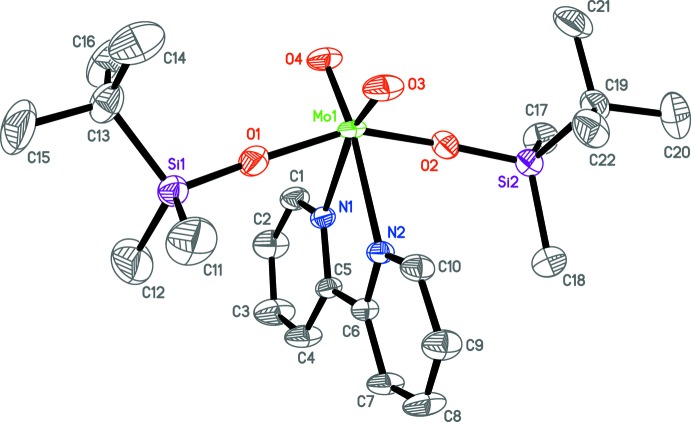
The mol­ecular structure of the title complex (I). Displacement ellipsoids are drawn at the 50% probability level and, for clarity, H atoms have been omitted.

A view of the mol­ecular structure of the 16-electron complex (^*t*^BuSiMe_2_O)_2_MoO_2_(bipy) (I) is given in Fig. 2 ▸, and selected geometrical parameters are given in Table 1 ▸. The bipy ligand is not planar, but instead twisted about the C5—C6 bond with a dihedral angle of 9.76 (14)° between the two pyridine rings. The Mo environment resembles a distorted octa­hedron with the bulky sil­oxy ligands occupying the *trans* positions. The *X*—Mo—*X* bond angles lie in the ranges 77.30 (7)–79.91 (7)° for O_Si_—Mo1—N, 97.22 (8)–98.38 (9) for O_Si_—Mo1—O_Mo=O_, 90.23 (7)–94.24 (7) for O_Mo=O_—Mo1—N (*cis*) and 159.26 (7)–163.31 (7) for O_Mo=O_—Mo1—N (*trans*). The Mo=O double bonds are, as expected, shorter by *ca* 0.20 Å than the Mo—O_Si_ single bonds (Table 1 ▸), while the Mo—N bond lengths are the longest.

The Mo—*X* bond lengths in five known complexes of types (Ph_3_SiO)_2_MoO_2_(*L*) and (Ph_3_SiO)_2_MoO_2_(py)_2_ (where *L* is a κ^2^ *N*,*N*′-coordinated ligand, py is pyridine; CSD refcodes are LEKCEL, SOKPAK, WIXCEL, WIXCIP and ZASHAE; see *Section 4, Database survey* below) vary from 1.695 to 1.705 Å for Mo=O, 1.923 to 1.939 Å for Mo—O_Si_ and 2.336–2.407 Å for Mo—N. Slightly shorter Mo—O bond lengths are found in the complexes (Ph_3_SiO)_2_MoO_2_(PPh_3_) (PERGAU; 1.678 and 1.678 Å for Mo=O, 1.903 and 1.922 Å for Mo—O_Si_) and (Ph_3_SiO)_2_MoO_2_ (PERFUN; 1.690 Å for Mo=O and 1.816 Å for Mo—O_Si_), likely because of the reduced number of coordinated σ-donating atoms. The title complex exhibits similar Mo=O and Mo—N bond lengths to those in (Ph_3_SiO)_2_MoO_2_(*L*), but the Mo—O_Si_ bond lengths are shorter by *ca* 0.02 Å, probably as a result of the lower steric influence of the ^*t*^BuSiMe_2_O ligand than that of Ph_3_SiO. The *X*—Mo—*X* bond angles in (I) and those in (Ph_3_SiO)_2_MoO_2_(*L*) are also similar.

## Supra­molecular features   

**Figure 3:**
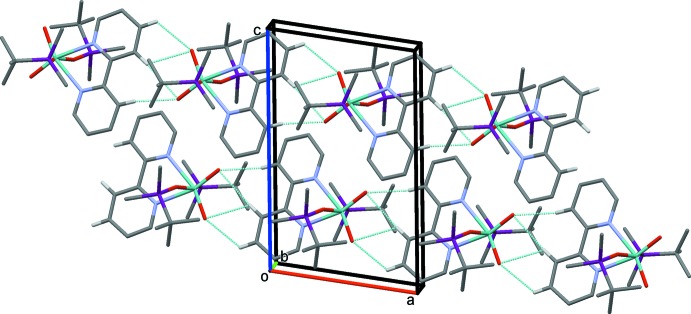
A view along the *b* axis of the crystal packing of the title complex (I). Only the H atoms involved in hydrogen bonding (dashed lines; see Table 2 ▸) are included.

In the crystal, neighbouring mol­ecules are linked by C—H⋯O=Mo hydrogen bonds, forming chains along the *a-*axis direction (Fig. 3 ▸ and Table 2 ▸). Similar Mo=O⋯H_Ar_ inter­actions can be found in the (Ph_3_SiO)_2_MoO_2_(*L*) complexes mentioned above. Other non-valent inter­molecular short contacts present in the structure of (I) are less significant.

## Database survey   

Crystal structures possessing the (*R*_3_SiO)_2_ *M*(=O)_2_ structural motif (*M* = Cr, Mo or W; *R* is alk­yl/ar­yl) are quite rare. Nine such structures have been described to date in the Cambridge Structural Database (CSD Version 5.39, latest update February 2018; Groom *et al.*, 2016 ▸), which have only *R* = Ph. There are two complexes of the type (Ph_3_SiO)_2_ *M*O_2_ without additional σ-donors (*M* = Mo, CSD refcode PERFUN: Huang & DeKock, 1993 ▸; *M* = Cr, PSILCR: Stensland & Kierkegaard, 1970 ▸), two complexes with σ-donating monodentate ligands, *viz*. (Ph_3_SiO)_2_MoO_2_(PPh_3_) (PERGAU: Huang & DeKock, 1993 ▸), (Ph_3_SiO)_2_MoO_2_(py)_2_ (py mol­ecules *cis*; WIXCIP: Thapper *et al.*, 1999 ▸) and five (Ph_3_SiO)_2_ *M*O_2_(*L*) complexes (where *M* = Mo and W; L is a κ^2^ *N*,*N*′-bidentate ligand). They include (Ph_3_SiO)_2_MoO_2_(bipy) (LEKCEL: Heppekausen *et al.*, 2012 ▸), (Ph_3_SiO)_2_MoO_2_(4,4′-^*t*^Bu_2_bipy) (SOKPAK: Arzoumanian *et al.*, 2008 ▸), (Ph_3_SiO)_2_MoO_2_(phen) (phen is 1,10-phenanthroline; WIXCEL: Thapper *et al.*, 1999 ▸), (Ph_3_SiO)_2_WO_2_(3,4,7,8-Me_4_phen)(MELGEP: Miao *et al.*, 2000 ▸) and (Ph_3_SiO)_2_MoO_2_(pzpy) (pzpy is 2-(1*H*-pyrazol-3-yl)pyridine; ZASHAE: Coelho *et al.*, 2011 ▸).

## Synthesis and crystallization   

The title Mo^VI^ complex was synthesized by a modification of previously reported methods for an analogous complex (Huang & DeKock, 1993 ▸; Bruno *et al.*, 2006 ▸). Details of the synthesis are illustrated in Fig. 1 ▸. Under an argon atmosphere, a stirred mixture of anhydrous sodium molybdate (0.41 g, 2.0 mmol) and 2,2-bi­pyridine (0.310 g, 2.0 mmol) in CH_3_CN (15 ml) was cooled to 273 K and a solution of *tert*-butyl­dimethyl­silyl chloride (0.603 g, 4.00 mmol) in CH_3_CN (10 ml) was slowly added. The obtained suspension was allowed to warm slowly to room temperature and was stirred overnight. All volatiles were removed under reduced pressure. The residue was extracted with THF (50 ml) and filtered. The filtrates were concentrated and cooled to 248 K to afford colourless crystals of (I) (yield 0.850 g, 1.55 mmol, 78%).

^1^H NMR (CD_2_Cl_2_, 298K) δ: −0.45 (*s*, 12H), 0.55 (*s*, 18H), 7.60 (*t*, 2H), 8.08 (*t*, 2H), 8.19 (*m*, 2H), 8.29 (*d*, 2H). ^13^C{^1^H} NMR (CD_2_Cl_2_, 298K) δ: −4.3, 19.5, 25.9, 122.0, 126.1, 139.8, 150.9. See the *Supporting information* for ^1^H and ^13^C{^1^H} NMR spectra. Analysis found (calculated for C_22_H_38_MoN_2_O_4_Si_2_): C 48.65 (48.33), H 7.30 (7.01), N 5.28% (5.12%).

## Refinement   

Crystal data, data collection and structure refinement details are summarized in Table 3 ▸. All H atoms were found from difference-Fourier maps but positioned geometrically and refined as riding: C—H = 0.95–0.98 Å with *U*_iso_(H) = 1.5*U*_eq_(C-meth­yl) and 1.2*U*_eq_(C) for other H atoms. A rotating group model was applied for the methyl groups. Reflections 001, 010 and 0
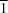
1 were omitted from the refinement as they were affected by the beam stop.

## Supplementary Material

Crystal structure: contains datablock(s) I, Global. DOI: 10.1107/S2056989018008472/su5447sup1.cif

Structure factors: contains datablock(s) I. DOI: 10.1107/S2056989018008472/su5447Isup2.hkl

Supporting information file. DOI: 10.1107/S2056989018008472/su5447Isup3.cdx

NMR spectra. DOI: 10.1107/S2056989018008472/su5447sup4.pdf

CCDC reference: 1833969

Additional supporting information:  crystallographic information; 3D view; checkCIF report

## Figures and Tables

**Table 1:** Selected geometric parameters (Å, °)

Mo1—O1	1.9001 (17)	Mo1—O4	1.7073 (16)
Mo1—O2	1.9149 (17)	Mo1—N1	2.3508 (18)
Mo1—O3	1.7058 (17)	Mo1—N2	2.3523 (18)
			
O1—Mo1—O2	153.41 (7)	O3—Mo1—N1	159.26 (7)
O1—Mo1—O3	98.38 (9)	O4—Mo1—N1	94.24 (7)
O1—Mo1—O4	97.22 (8)	O1—Mo1—N2	79.53 (7)
O2—Mo1—O3	98.38 (8)	O2—Mo1—N2	79.91 (7)
O2—Mo1—O4	97.63 (8)	O3—Mo1—N2	90.23 (7)
O3—Mo1—O4	106.46 (8)	O4—Mo1—N2	163.31 (7)
O1—Mo1—N1	77.30 (7)	N1—Mo1—N2	69.07 (6)
O2—Mo1—N1	79.72 (7)		

**Table 2:** Hydrogen-bond geometry (Å, °)

*D*—H⋯*A*	*D*—H	H⋯*A*	*D*⋯*A*	*D*—H⋯*A*
C4—H4⋯O4^i^	0.95	2.38	3.260 (3)	153
C7—H7⋯O3^i^	0.95	2.59	3.189 (3)	122
C7—H7⋯O4^i^	0.95	2.55	3.494 (3)	170
C8—H8⋯O3^i^	0.95	2.55	3.168 (3)	123

Symmetry code: (i) 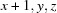.

**Table 3:** Experimental details

Crystal data	
Chemical formula	[Mo(C_6_H_15_OSi)_2_O_2_(C_10_H_8_N_2_)]
*M*_r_	546.66
Crystal system, space group	Triclinic, *P* 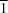
Temperature (K)	150
*a*, *b*, *c* (Å)	8.4027 (8), 12.8657 (13), 14.4266 (14)
α, β, γ (°)	113.144 (2), 91.133 (2), 105.501 (2)
*V* (Å^3^)	1368.1 (2)
*Z*	2
Radiation type	Mo *K*α
μ (mm^−1^)	0.59
Crystal size (mm)	0.37 × 0.16 × 0.01

Data collection	
Diffractometer	Bruker SMART APEXII
Absorption correction	Multi-scan (*SADABS*; Bruker, 2008 ▸)
*T*_min_, *T*_max_	0.612, 0.797
No. of measured, independent and observed [*I* > 2σ(*I*)] reflections	14073, 6550, 5158
*R*_int_	0.030
(sin θ/λ)_max_ (Å^−1^)	0.660

Refinement	
*R*[*F* ^2^ > 2σ(*F* ^2^)], *wR*(*F* ^2^), *S*	0.034, 0.075, 1.01
No. of reflections	6550
No. of parameters	290
H-atom treatment	H-atom parameters constrained
Δρ_max_, Δρ_min_ (e Å^−3^)	0.50, −0.76

Computer programs: *APEX2* and *SAINT* (Bruker, 2008 ▸), *SHELXS* and *SHELXTL* (Sheldrick, 2008 ▸), *SHELXL2017/1* (Sheldrick, 2015 ▸), *Mercury* (Macrae *et al.*, 2008 ▸), and *publCIF* (Westrip, 2010 ▸).

## supplementary crystallographic information

### Crystal data

**Table:**

[Mo(C_6_H_15_OSi)_2_O_2_(C_10_H_8_N_2_)]	*Z* = 2
*M**_r_* = 546.66	*F*(000) = 572
Triclinic, *P*1	*D*_x_ = 1.327 Mg m^−^^3^
*a* = 8.4027 (8) Å	Mo *K*α radiation, λ = 0.71073 Å
*b* = 12.8657 (13) Å	Cell parameters from 4261 reflections
*c* = 14.4266 (14) Å	θ = 2.5–30.2°
α = 113.144 (2)°	µ = 0.59 mm^−^^1^
β = 91.133 (2)°	*T* = 150 K
γ = 105.501 (2)°	Plate, colourless
*V* = 1368.1 (2) Å^3^	0.37 × 0.16 × 0.01 mm

### Data collection

**Table:**

Bruker SMART APEXII diffractometer	6550 independent reflections
Radiation source: fine-focus sealed tube	5158 reflections with *I* > 2σ(*I*)
Graphite monochromator	*R*_int_ = 0.030
ω scans	θ_max_ = 28.0°, θ_min_ = 2.5°
Absorption correction: multi-scan (SADABS; Bruker, 2008)	*h* = −11→11
*T*_min_ = 0.612, *T*_max_ = 0.797	*k* = −16→16
14073 measured reflections	*l* = −19→19

### Refinement

**Table:**

Refinement on *F*^2^	Primary atom site location: structure-invariant direct methods
Least-squares matrix: full	Secondary atom site location: difference Fourier map
*R*[*F*^2^ > 2σ(*F*^2^)] = 0.034	Hydrogen site location: inferred from neighbouring sites
*wR*(*F*^2^) = 0.075	H-atom parameters constrained
*S* = 1.01	*w* = 1/[σ^2^(*F*_o_^2^) + (0.0328*P*)^2^ + 0.2626*P*] where *P* = (*F*_o_^2^ + 2*F*_c_^2^)/3
6550 reflections	(Δ/σ)_max_ = 0.001
290 parameters	Δρ_max_ = 0.50 e Å^−^^3^
0 restraints	Δρ_min_ = −0.76 e Å^−^^3^

### Special details

**Table:**

Experimental. All synthetic manipulations were conducted under an argon atmosphere, using a dry box and standard Schlenk and vacuum line techniques. THF was predried over NaOH and distilled from potassium/benzophenoneketyl under argon. CH3CN was distilled from calcium hydride under argon. CD2Cl2 was carefully distilled from LiAlH4 and stored over 4?Å molecular sieves. The Mo complex was synthesized by a modification of previously reported methods for an analogous complex (Huang & DeKock, 1993; Bruno *et al.*, 2006). Elemental (C, H, N) analysis was performed with a PerkinElmer 2400 Series II elemental CHNS/O analyzer. NMR spectra were recorded with a Bruker AVANCE 400 spectrometer at 298K.
Geometry. All esds (except the esd in the dihedral angle between two l.s. planes) are estimated using the full covariance matrix. The cell esds are taken into account individually in the estimation of esds in distances, angles and torsion angles; correlations between esds in cell parameters are only used when they are defined by crystal symmetry. An approximate (isotropic) treatment of cell esds is used for estimating esds involving l.s. planes.
Refinement. Refinement of F^2^ against ALL reflections. The weighted R-factor wR and goodness of fit S are based on F^2^, conventional R-factors R are based on F, with F set to zero for negative F^2^. The threshold expression of F^2^ > 2sigma(F^2^) is used only for calculating R-factors(gt) etc. and is not relevant to the choice of reflections for refinement. R-factors based on F^2^ are statistically about twice as large as those based on F, and R- factors based on ALL data will be even larger.

### Fractional atomic coordinates and isotropic or equivalent isotropic displacement parameters (Å^2^)

**Table:**

	*x*	*y*	*z*	*U*_iso_*/*U*_eq_	
Mo1	0.52976 (2)	0.50708 (2)	0.72617 (2)	0.02159 (6)	
Si1	0.49782 (9)	0.23030 (6)	0.72652 (5)	0.03018 (16)	
Si2	0.76259 (8)	0.79213 (6)	0.75979 (5)	0.02501 (15)	
N1	0.7113 (2)	0.44628 (16)	0.60828 (14)	0.0215 (4)	
N2	0.7921 (2)	0.53749 (17)	0.80986 (14)	0.0212 (4)	
O1	0.5091 (2)	0.35145 (15)	0.71423 (14)	0.0351 (4)	
O2	0.6496 (2)	0.65577 (14)	0.72517 (12)	0.0294 (4)	
O3	0.4593 (2)	0.55966 (17)	0.84064 (13)	0.0349 (4)	
O4	0.36694 (19)	0.46985 (16)	0.63499 (13)	0.0329 (4)	
C1	0.6609 (3)	0.3972 (2)	0.50836 (18)	0.0292 (5)	
H1	0.555036	0.397932	0.484733	0.035*	
C2	0.7559 (3)	0.3452 (3)	0.43764 (19)	0.0398 (7)	
H2	0.716631	0.310979	0.366791	0.048*	
C3	0.9084 (3)	0.3443 (3)	0.4724 (2)	0.0458 (8)	
H3	0.975418	0.307597	0.425645	0.055*	
C4	0.9640 (3)	0.3968 (2)	0.57561 (19)	0.0350 (6)	
H4	1.070137	0.397791	0.600557	0.042*	
C5	0.8626 (3)	0.4483 (2)	0.64229 (17)	0.0220 (5)	
C6	0.9120 (3)	0.5063 (2)	0.75423 (17)	0.0213 (5)	
C7	1.0721 (3)	0.5303 (2)	0.79989 (18)	0.0302 (6)	
H7	1.155480	0.508329	0.759516	0.036*	
C8	1.1091 (3)	0.5865 (3)	0.90479 (19)	0.0365 (7)	
H8	1.218304	0.604139	0.937403	0.044*	
C9	0.9851 (3)	0.6166 (2)	0.96145 (19)	0.0335 (6)	
H9	1.007172	0.654825	1.033667	0.040*	
C10	0.8284 (3)	0.5901 (2)	0.91123 (17)	0.0266 (5)	
H10	0.742823	0.609994	0.950338	0.032*	
C11	0.5902 (4)	0.2679 (3)	0.8581 (3)	0.0663 (10)	
H11A	0.540588	0.323552	0.907140	0.099*	
H11B	0.711021	0.304435	0.866646	0.099*	
H11C	0.567837	0.195617	0.869773	0.099*	
C12	0.6180 (5)	0.1462 (3)	0.6336 (3)	0.0723 (11)	
H12A	0.735789	0.193257	0.648619	0.108*	
H12B	0.573603	0.130178	0.564370	0.108*	
H12C	0.607803	0.071187	0.638913	0.108*	
C13	0.2713 (4)	0.1436 (3)	0.7013 (2)	0.0406 (7)	
C14	0.1814 (4)	0.2076 (4)	0.7858 (3)	0.0682 (11)	
H14A	0.062089	0.164346	0.770465	0.102*	
H14B	0.197187	0.287983	0.790677	0.102*	
H14C	0.227105	0.211773	0.850591	0.102*	
C15	0.2504 (5)	0.0183 (3)	0.6950 (3)	0.0739 (12)	
H15A	0.131507	−0.026089	0.680629	0.111*	
H15B	0.298124	0.024024	0.760015	0.111*	
H15C	0.308173	−0.022823	0.640459	0.111*	
C16	0.1928 (4)	0.1339 (3)	0.6008 (3)	0.0663 (11)	
H16A	0.073361	0.091631	0.589027	0.099*	
H16B	0.246525	0.090336	0.545051	0.099*	
H16C	0.208028	0.213434	0.603852	0.099*	
C17	0.7485 (4)	0.8299 (3)	0.6479 (2)	0.0395 (7)	
H17A	0.779816	0.771938	0.589129	0.059*	
H17B	0.824427	0.909248	0.664271	0.059*	
H17C	0.633928	0.828417	0.631510	0.059*	
C18	0.9844 (3)	0.8106 (2)	0.8014 (2)	0.0341 (6)	
H18A	1.028364	0.758651	0.745049	0.051*	
H18B	0.989910	0.789604	0.859472	0.051*	
H18C	1.051098	0.893156	0.821405	0.051*	
C19	0.6780 (3)	0.8919 (2)	0.86890 (19)	0.0323 (6)	
C20	0.7706 (4)	1.0223 (3)	0.8951 (2)	0.0536 (8)	
H20A	0.729747	1.073145	0.953875	0.080*	
H20B	0.750759	1.040231	0.836610	0.080*	
H20C	0.890478	1.036863	0.911493	0.080*	
C21	0.4918 (4)	0.8679 (3)	0.8387 (2)	0.0483 (8)	
H21A	0.447629	0.919161	0.895310	0.072*	
H21B	0.432569	0.784734	0.822421	0.072*	
H21C	0.475998	0.884299	0.778858	0.072*	
C22	0.6998 (4)	0.8661 (3)	0.96252 (19)	0.0405 (7)	
H22A	0.657102	0.918716	1.019096	0.061*	
H22B	0.818368	0.879441	0.981903	0.061*	
H22C	0.637747	0.783454	0.946496	0.061*	

### Atomic displacement parameters (Å^2^)

**Table:**

	*U*^11^	*U*^22^	*U*^33^	*U*^12^	*U*^13^	*U*^23^
Mo1	0.01231 (9)	0.02799 (12)	0.02539 (11)	0.00788 (7)	0.00225 (7)	0.01080 (9)
Si1	0.0280 (4)	0.0293 (4)	0.0335 (4)	0.0066 (3)	0.0005 (3)	0.0146 (3)
Si2	0.0272 (3)	0.0270 (4)	0.0230 (3)	0.0095 (3)	0.0036 (3)	0.0115 (3)
N1	0.0166 (9)	0.0228 (10)	0.0218 (10)	0.0062 (8)	0.0006 (7)	0.0058 (8)
N2	0.0171 (9)	0.0242 (10)	0.0222 (10)	0.0083 (8)	0.0025 (7)	0.0081 (8)
O1	0.0257 (9)	0.0324 (10)	0.0488 (12)	0.0036 (8)	0.0011 (8)	0.0218 (9)
O2	0.0305 (9)	0.0267 (9)	0.0312 (10)	0.0125 (8)	0.0051 (7)	0.0096 (8)
O3	0.0221 (9)	0.0522 (12)	0.0308 (10)	0.0149 (8)	0.0078 (7)	0.0148 (9)
O4	0.0193 (8)	0.0445 (11)	0.0343 (10)	0.0114 (8)	−0.0014 (7)	0.0147 (9)
C1	0.0229 (12)	0.0337 (14)	0.0254 (13)	0.0094 (11)	−0.0037 (10)	0.0062 (11)
C2	0.0383 (15)	0.0523 (18)	0.0196 (13)	0.0182 (14)	0.0001 (11)	0.0027 (12)
C3	0.0340 (15)	0.067 (2)	0.0266 (14)	0.0262 (15)	0.0069 (11)	0.0026 (14)
C4	0.0223 (12)	0.0529 (18)	0.0266 (13)	0.0184 (12)	0.0041 (10)	0.0086 (12)
C5	0.0152 (10)	0.0256 (13)	0.0236 (12)	0.0063 (9)	0.0022 (9)	0.0084 (10)
C6	0.0178 (10)	0.0255 (12)	0.0213 (11)	0.0081 (9)	0.0023 (9)	0.0094 (10)
C7	0.0190 (11)	0.0462 (16)	0.0267 (13)	0.0161 (11)	0.0041 (9)	0.0123 (12)
C8	0.0219 (12)	0.0586 (19)	0.0277 (14)	0.0165 (12)	−0.0023 (10)	0.0139 (13)
C9	0.0312 (13)	0.0473 (17)	0.0206 (12)	0.0160 (12)	−0.0001 (10)	0.0102 (12)
C10	0.0240 (12)	0.0349 (14)	0.0228 (12)	0.0140 (11)	0.0064 (9)	0.0104 (11)
C11	0.064 (2)	0.074 (3)	0.059 (2)	0.0102 (19)	−0.0221 (18)	0.034 (2)
C12	0.068 (2)	0.055 (2)	0.093 (3)	0.029 (2)	0.037 (2)	0.022 (2)
C13	0.0400 (15)	0.0408 (17)	0.0405 (16)	−0.0009 (13)	−0.0004 (12)	0.0249 (14)
C14	0.0435 (19)	0.092 (3)	0.070 (2)	0.0143 (19)	0.0225 (18)	0.038 (2)
C15	0.065 (2)	0.053 (2)	0.103 (3)	−0.0100 (18)	0.003 (2)	0.050 (2)
C16	0.054 (2)	0.070 (2)	0.057 (2)	−0.0134 (18)	−0.0222 (17)	0.0308 (19)
C17	0.0412 (15)	0.0485 (18)	0.0363 (16)	0.0132 (14)	0.0044 (12)	0.0252 (14)
C18	0.0304 (13)	0.0425 (16)	0.0315 (14)	0.0087 (12)	0.0056 (11)	0.0188 (12)
C19	0.0362 (14)	0.0296 (14)	0.0300 (14)	0.0148 (12)	0.0033 (11)	0.0080 (11)
C20	0.077 (2)	0.0313 (17)	0.0459 (19)	0.0173 (16)	0.0028 (16)	0.0084 (14)
C21	0.0469 (17)	0.054 (2)	0.0408 (17)	0.0332 (16)	0.0069 (13)	0.0051 (15)
C22	0.0516 (17)	0.0431 (17)	0.0249 (14)	0.0232 (14)	0.0093 (12)	0.0062 (12)

### Geometric parameters (Å, º)

**Table:**

Mo1—O1	1.9001 (17)	C11—H11B	0.9800
Mo1—O2	1.9149 (17)	C11—H11C	0.9800
Mo1—O3	1.7058 (17)	C12—H12A	0.9800
Mo1—O4	1.7073 (16)	C12—H12B	0.9800
Mo1—N1	2.3508 (18)	C12—H12C	0.9800
Mo1—N2	2.3523 (18)	C13—C14	1.521 (5)
Si1—O1	1.6152 (18)	C13—C16	1.525 (4)
Si1—C11	1.860 (3)	C13—C15	1.540 (4)
Si1—C12	1.867 (3)	C14—H14A	0.9800
Si1—C13	1.876 (3)	C14—H14B	0.9800
Si2—O2	1.6219 (18)	C14—H14C	0.9800
Si2—C18	1.870 (3)	C15—H15A	0.9800
Si2—C17	1.871 (3)	C15—H15B	0.9800
Si2—C19	1.891 (3)	C15—H15C	0.9800
N1—C1	1.332 (3)	C16—H16A	0.9800
N1—C5	1.344 (3)	C16—H16B	0.9800
N2—C10	1.335 (3)	C16—H16C	0.9800
N2—C6	1.347 (3)	C17—H17A	0.9800
C1—C2	1.381 (3)	C17—H17B	0.9800
C1—H1	0.9500	C17—H17C	0.9800
C2—C3	1.372 (4)	C18—H18A	0.9800
C2—H2	0.9500	C18—H18B	0.9800
C3—C4	1.380 (3)	C18—H18C	0.9800
C3—H3	0.9500	C19—C21	1.530 (4)
C4—C5	1.387 (3)	C19—C22	1.531 (4)
C4—H4	0.9500	C19—C20	1.535 (4)
C5—C6	1.482 (3)	C20—H20A	0.9800
C6—C7	1.387 (3)	C20—H20B	0.9800
C7—C8	1.382 (3)	C20—H20C	0.9800
C7—H7	0.9500	C21—H21A	0.9800
C8—C9	1.380 (3)	C21—H21B	0.9800
C8—H8	0.9500	C21—H21C	0.9800
C9—C10	1.379 (3)	C22—H22A	0.9800
C9—H9	0.9500	C22—H22B	0.9800
C10—H10	0.9500	C22—H22C	0.9800
C11—H11A	0.9800		
			
O1—Mo1—O2	153.41 (7)	H11A—C11—H11C	109.5
O1—Mo1—O3	98.38 (9)	H11B—C11—H11C	109.5
O1—Mo1—O4	97.22 (8)	Si1—C12—H12A	109.5
O2—Mo1—O3	98.38 (8)	Si1—C12—H12B	109.5
O2—Mo1—O4	97.63 (8)	H12A—C12—H12B	109.5
O3—Mo1—O4	106.46 (8)	Si1—C12—H12C	109.5
O1—Mo1—N1	77.30 (7)	H12A—C12—H12C	109.5
O2—Mo1—N1	79.72 (7)	H12B—C12—H12C	109.5
O3—Mo1—N1	159.26 (7)	C14—C13—C16	108.3 (3)
O4—Mo1—N1	94.24 (7)	C14—C13—C15	109.6 (3)
O1—Mo1—N2	79.53 (7)	C16—C13—C15	109.2 (3)
O2—Mo1—N2	79.91 (7)	C14—C13—Si1	109.7 (2)
O3—Mo1—N2	90.23 (7)	C16—C13—Si1	109.4 (2)
O4—Mo1—N2	163.31 (7)	C15—C13—Si1	110.6 (2)
N1—Mo1—N2	69.07 (6)	C13—C14—H14A	109.5
O1—Si1—C11	108.92 (14)	C13—C14—H14B	109.5
O1—Si1—C12	109.23 (15)	H14A—C14—H14B	109.5
C11—Si1—C12	109.27 (19)	C13—C14—H14C	109.5
O1—Si1—C13	107.15 (11)	H14A—C14—H14C	109.5
C11—Si1—C13	111.06 (15)	H14B—C14—H14C	109.5
C12—Si1—C13	111.14 (16)	C13—C15—H15A	109.5
O2—Si2—C18	110.49 (11)	C13—C15—H15B	109.5
O2—Si2—C17	107.57 (11)	H15A—C15—H15B	109.5
C18—Si2—C17	110.27 (12)	C13—C15—H15C	109.5
O2—Si2—C19	109.25 (11)	H15A—C15—H15C	109.5
C18—Si2—C19	109.30 (12)	H15B—C15—H15C	109.5
C17—Si2—C19	109.94 (13)	C13—C16—H16A	109.5
C1—N1—C5	119.01 (19)	C13—C16—H16B	109.5
C1—N1—Mo1	121.42 (15)	H16A—C16—H16B	109.5
C5—N1—Mo1	119.10 (14)	C13—C16—H16C	109.5
C10—N2—C6	118.84 (19)	H16A—C16—H16C	109.5
C10—N2—Mo1	121.80 (14)	H16B—C16—H16C	109.5
C6—N2—Mo1	119.28 (14)	Si2—C17—H17A	109.5
Si1—O1—Mo1	169.53 (12)	Si2—C17—H17B	109.5
Si2—O2—Mo1	163.13 (11)	H17A—C17—H17B	109.5
N1—C1—C2	122.8 (2)	Si2—C17—H17C	109.5
N1—C1—H1	118.6	H17A—C17—H17C	109.5
C2—C1—H1	118.6	H17B—C17—H17C	109.5
C3—C2—C1	118.2 (2)	Si2—C18—H18A	109.5
C3—C2—H2	120.9	Si2—C18—H18B	109.5
C1—C2—H2	120.9	H18A—C18—H18B	109.5
C2—C3—C4	119.8 (2)	Si2—C18—H18C	109.5
C2—C3—H3	120.1	H18A—C18—H18C	109.5
C4—C3—H3	120.1	H18B—C18—H18C	109.5
C3—C4—C5	118.9 (2)	C21—C19—C22	108.6 (2)
C3—C4—H4	120.5	C21—C19—C20	109.5 (2)
C5—C4—H4	120.5	C22—C19—C20	109.4 (2)
N1—C5—C4	121.3 (2)	C21—C19—Si2	109.38 (18)
N1—C5—C6	116.11 (19)	C22—C19—Si2	109.81 (17)
C4—C5—C6	122.6 (2)	C20—C19—Si2	110.15 (19)
N2—C6—C7	121.4 (2)	C19—C20—H20A	109.5
N2—C6—C5	115.85 (18)	C19—C20—H20B	109.5
C7—C6—C5	122.7 (2)	H20A—C20—H20B	109.5
C8—C7—C6	119.3 (2)	C19—C20—H20C	109.5
C8—C7—H7	120.4	H20A—C20—H20C	109.5
C6—C7—H7	120.4	H20B—C20—H20C	109.5
C9—C8—C7	119.0 (2)	C19—C21—H21A	109.5
C9—C8—H8	120.5	C19—C21—H21B	109.5
C7—C8—H8	120.5	H21A—C21—H21B	109.5
C10—C9—C8	118.7 (2)	C19—C21—H21C	109.5
C10—C9—H9	120.6	H21A—C21—H21C	109.5
C8—C9—H9	120.6	H21B—C21—H21C	109.5
N2—C10—C9	122.7 (2)	C19—C22—H22A	109.5
N2—C10—H10	118.6	C19—C22—H22B	109.5
C9—C10—H10	118.6	H22A—C22—H22B	109.5
Si1—C11—H11A	109.5	C19—C22—H22C	109.5
Si1—C11—H11B	109.5	H22A—C22—H22C	109.5
H11A—C11—H11B	109.5	H22B—C22—H22C	109.5
Si1—C11—H11C	109.5		
			
C11—Si1—O1—Mo1	−16.0 (7)	N2—C6—C7—C8	0.5 (4)
C12—Si1—O1—Mo1	−135.2 (6)	C5—C6—C7—C8	178.9 (2)
C13—Si1—O1—Mo1	104.3 (6)	C6—C7—C8—C9	0.5 (4)
C18—Si2—O2—Mo1	68.6 (4)	C7—C8—C9—C10	−0.4 (4)
C17—Si2—O2—Mo1	−171.0 (3)	C6—N2—C10—C9	1.7 (4)
C19—Si2—O2—Mo1	−51.7 (4)	Mo1—N2—C10—C9	−175.1 (2)
C5—N1—C1—C2	1.7 (4)	C8—C9—C10—N2	−0.7 (4)
Mo1—N1—C1—C2	−170.4 (2)	O1—Si1—C13—C14	−67.7 (2)
N1—C1—C2—C3	0.1 (4)	C11—Si1—C13—C14	51.2 (3)
C1—C2—C3—C4	−1.4 (5)	C12—Si1—C13—C14	173.0 (2)
C2—C3—C4—C5	0.9 (5)	O1—Si1—C13—C16	51.0 (3)
C1—N1—C5—C4	−2.2 (4)	C11—Si1—C13—C16	169.8 (2)
Mo1—N1—C5—C4	170.10 (19)	C12—Si1—C13—C16	−68.3 (3)
C1—N1—C5—C6	178.9 (2)	O1—Si1—C13—C15	171.3 (2)
Mo1—N1—C5—C6	−8.9 (3)	C11—Si1—C13—C15	−69.9 (3)
C3—C4—C5—N1	0.9 (4)	C12—Si1—C13—C15	52.0 (3)
C3—C4—C5—C6	179.8 (3)	O2—Si2—C19—C21	−53.3 (2)
C10—N2—C6—C7	−1.6 (4)	C18—Si2—C19—C21	−174.34 (19)
Mo1—N2—C6—C7	175.28 (19)	C17—Si2—C19—C21	64.5 (2)
C10—N2—C6—C5	179.9 (2)	O2—Si2—C19—C22	65.7 (2)
Mo1—N2—C6—C5	−3.2 (3)	C18—Si2—C19—C22	−55.3 (2)
N1—C5—C6—N2	7.9 (3)	C17—Si2—C19—C22	−176.46 (19)
C4—C5—C6—N2	−171.0 (2)	O2—Si2—C19—C20	−173.69 (18)
N1—C5—C6—C7	−170.6 (2)	C18—Si2—C19—C20	65.3 (2)
C4—C5—C6—C7	10.4 (4)	C17—Si2—C19—C20	−55.9 (2)

### Hydrogen-bond geometry (Å, º)

**Table:**

*D*—H···*A*	*D*—H	H···*A*	*D*···*A*	*D*—H···*A*
C4—H4···O4^i^	0.95	2.38	3.260 (3)	153
C7—H7···O3^i^	0.95	2.59	3.189 (3)	122
C7—H7···O4^i^	0.95	2.55	3.494 (3)	170
C8—H8···O3^i^	0.95	2.55	3.168 (3)	123
C10—H10···O3	0.95	2.61	3.118 (3)	114

Symmetry code: (i) *x*+1, *y*, *z*.
