# Supplementary material for: Crystal structure of (2,2′-bi­pyridine-κ2 N,N′)-trans-bis­(tert-butyl­dimethyl­sil­yloxy)-cis-dioxidomolybdenum(VI)
Source: Acta Crystallogr E Crystallogr Commun. 2018 Jun 15;74(Pt 7):970–2. doi: 10.1107/S2056989018008472 (PMC6038637; doi:10.1107/S2056989018008472)
Supplement: Supplementary file 4 [file e-74-00970-sup4.pdf]

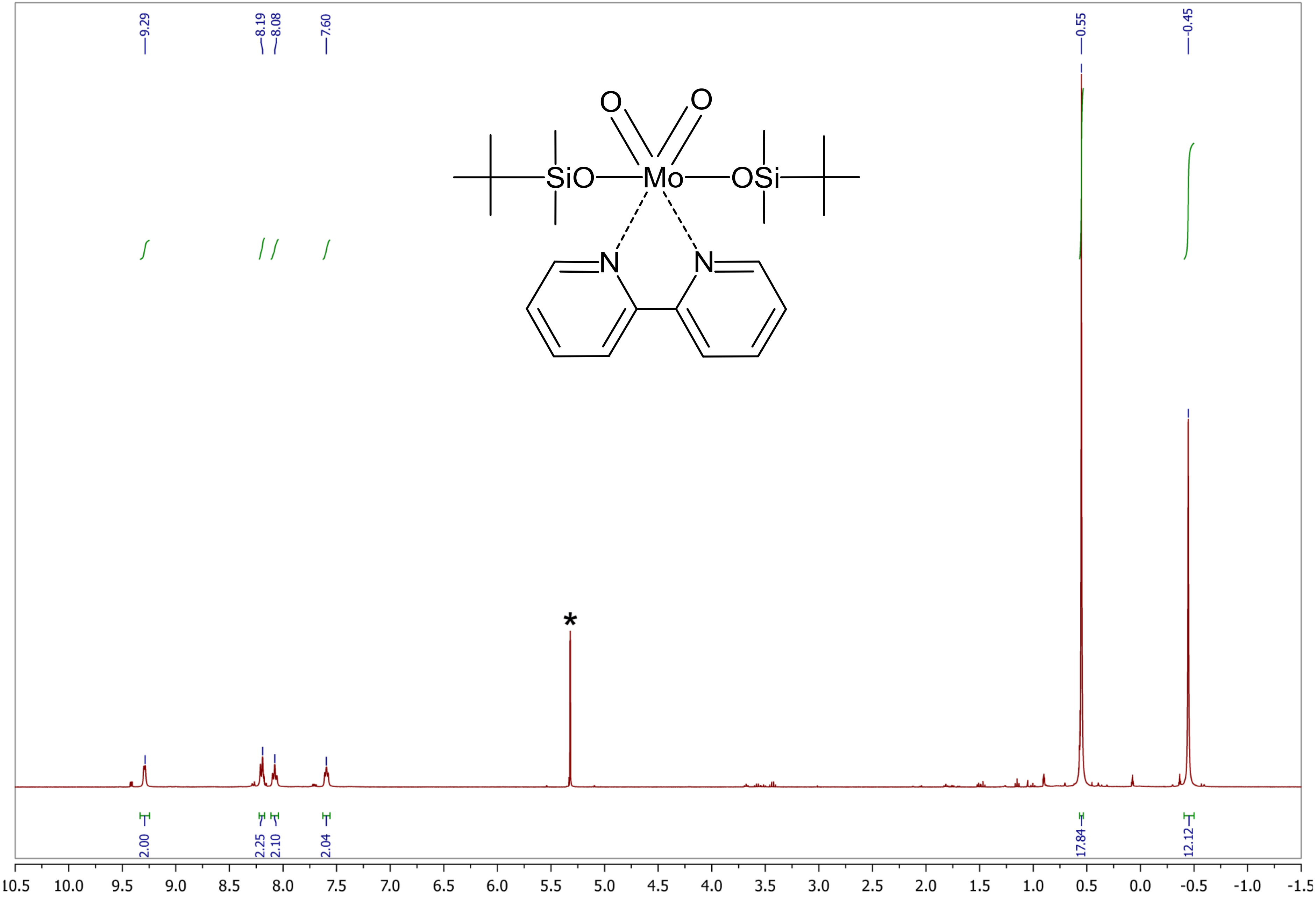

^{1}H NMR spectrum of [(^{t}BuSiMe$_2$O)$_2$MoO$_2$(bipy)] in CD_2Cl_2 at 400MHz.

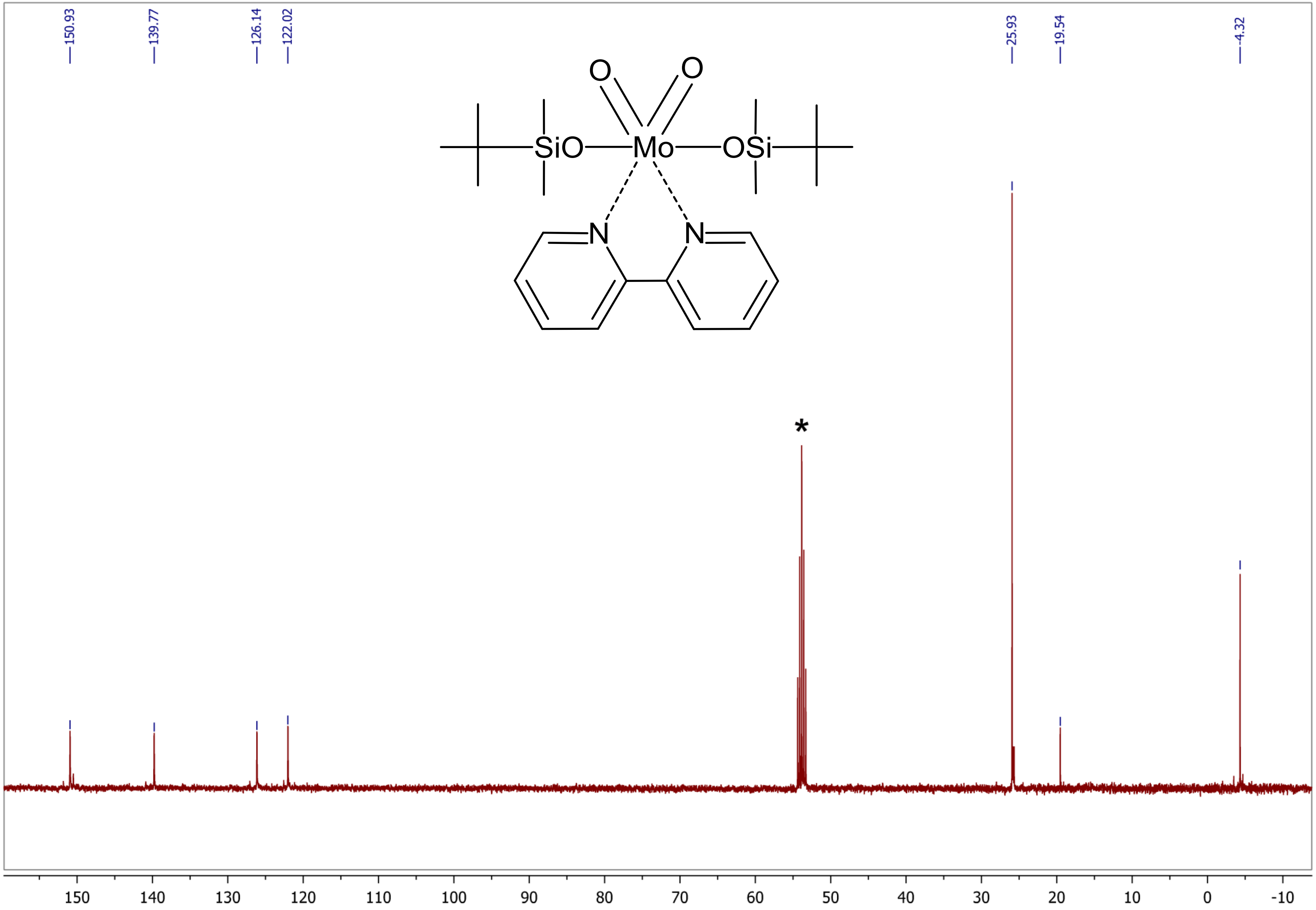

$^{13}C\{^{1}H\}$ NMR spectrum of [($^{t}BuSiMe_2O)_2MoO_2$(bipy)] in CD_2Cl_2 at 100MHz.
